# TMX family genes and their association with prognosis, immune infiltration, and chemotherapy in human pan-cancer

**DOI:** 10.18632/aging.205332

**Published:** 2023-12-21

**Authors:** Na Luo, Zhiqiang Mei, Qiqi Zhang, Hong Tang, Runlan Wan, Anni Deng, Xiaopan Zou, Chaoxiang Lv

**Affiliations:** 1 The Research Center for Preclinical Medicine, Southwest Medical University, Luzhou 646000, Sichuan, China; 2 School of Basic Medical Sciences, Southwest Medical University, Luzhou 646000, Sichuan, China; 3 Degree Office, The Graduate School of Southwest Medical University, Luzhou 646000, Sichuan, China; 4 Department of Pathology, Affiliated Hospital of Southwest Medical University, Luzhou 646000, Sichuan, China; 5 Department of Oncology, The Affiliated Hospital of Southwest Medical University, Luzhou 646000, Sichuan, China; 6 Department of Pediatrics, Southwest Medical University, Luzhou 646000, Sichuan, China; 7 Breast and Thyroid Surgery, Renmin Hospital, Jilin University, Changchun 130024, Jilin, China

**Keywords:** pan-cancer, thioredoxin, tumor microenvironment, immune infiltration subtypes, epithelial-mesenchymal transition

## Abstract

Background: The thioredoxin (TMX) system, an important redox system, plays crucial roles in several immune-related diseases. However, there is limited research on the correlation of TMX family gene expression with human pan-cancer prognosis, tumor microenvironment (TME), and immunotherapy.

Methods: Based on the integration of several bioinformatics analysis methods, we explored the expression levels and prognostic value of TMX family members in pan-cancer and analyzed their association between TME, immune infiltration, stemness scores, and drug sensitivity. Using KEGG enrichment analysis, we explored the potential signaling pathways of their regulation. Additionally, we conducted a transwell assay to verify the relationship between TMX family gene expression and epithelial–mesenchymal transition (EMT) in liver cancer.

Results: Expression of the TMX family genes was shown to have an obvious intratumoral heterogeneity. In some cancers, TMX family members expression was also been found to correlate with poor prognosis of patients. Furthermore, TMX family genes may serve important roles in TME. The expression of TMX family genes was found to have a strong correlation with the stromal scores, immune scores, DNAss and RNAss in pan-cancer. Specifically, the expression levels of TMX family genes have been found to be associated with immune subtypes of renal clear cell carcinoma and liver hepatocellular carcinoma. High *TMX2* expression promote EMT in liver cancer.

Conclusions: The findings of this study may elucidate the biological roles of TMX family genes as potential targets for pan-cancer and also offer valuable insights for further investigating how these genes function in the development and spreading of cancer.

## INTRODUCTION

The thioredoxin system is an important oxidation reduction system *in vivo*, including thioredoxin (TMX), glutaredoxin, thioredoxin reductase, nicotinamide adenine dinucleotide phosphate, thioredoxin peroxidase, and so on [1–3]. This system is widespread in mammalian cells and has several important functions such as anti-oxidation, anti-apoptosis and transcriptional regulation [4, 5]. Recently, the TMX system was found to as a crucial role during cancer initiation, progression and even deterioration [6]. It has been identified in various tumor cells, such as gastric cancer [7], colorectal cancer [8], liver cancer [9], breast cancer [10] and pancreatic cancer [11]. Thus, further exploration of physiological functions of TMX system and its molecular mechanism participating in tumor progression can raise the comprehension of tumor biology and offer the foundation for the discovery of new cancer therapeutic molecules.

TMX family genes mainly include *TMX1*, *TMX2*, *TMX3* and *TMX4*. They are usually located in the endoplasmic reticulum and act as protein disulfide isomerases that catalyze mismatched RNases to restore their normal structure [12]. TMX family genes play main roles in the folding of proteins and can be expressed in multiple cells. Previous studies have shown that TMX family genes are associated with various immune-related diseases [5]. For example, *TMX1* expression is associated with properties of tumor growth, and it has been proposed as a candidate antioncogene for breast cancer [13]. The inhibition of *TMX2* expression decreased cell proliferation of breast cancer and also resulted in a reduction in the expression of genes related to the survival, differentiation, and metastasis of cancer cells [14]. In addition, *TMX1*, *TMX2*, and *TMX3* are highly expressed in liver cancer cell lines, while *TMX4* expressed is upregulated in melanoma cell lines [15]. It means that the expression of the TMX family genes have an obvious intratumoral heterogeneity. *TMX2* interference attenuates mitochondrial respiration, leading to enhanced aerobic glycolysis, which promotes tumor cell growth [16]. Although the roles of TMX family genes in cancer is increasingly discovered, their biological roles in tumor immune response and the association of their expression with immunotherapy drug resistance are seldom reported. In particular, we lack knowledge about the function and clinical significance of TMX family genes in the context of human pan-cancer. Therefore, a systematic study is needed to comprehensively explore these aspects.

Here, we deeply explored the expression features of TMX family genes by using multi-omics cancer data and evaluated their expression levels in relation to the immune system, TME, tumor stemness. Besides, we analyzed the possible contribution of TMX family members to immunotherapy of drug resistance. Simultaneously, we discovered the correlation of their expression with genes associated immune checkpoints and tumor metastasis. These findings highlight the multifaceted roles of TMX family genes in various human cancers, offering a valuable theoretical foundation and potential targets for further investigation of their functions in cancer treatment.

## RESULTS

### The expression patterns of TMX family genes in pan-cancer data

**Figure 1:**
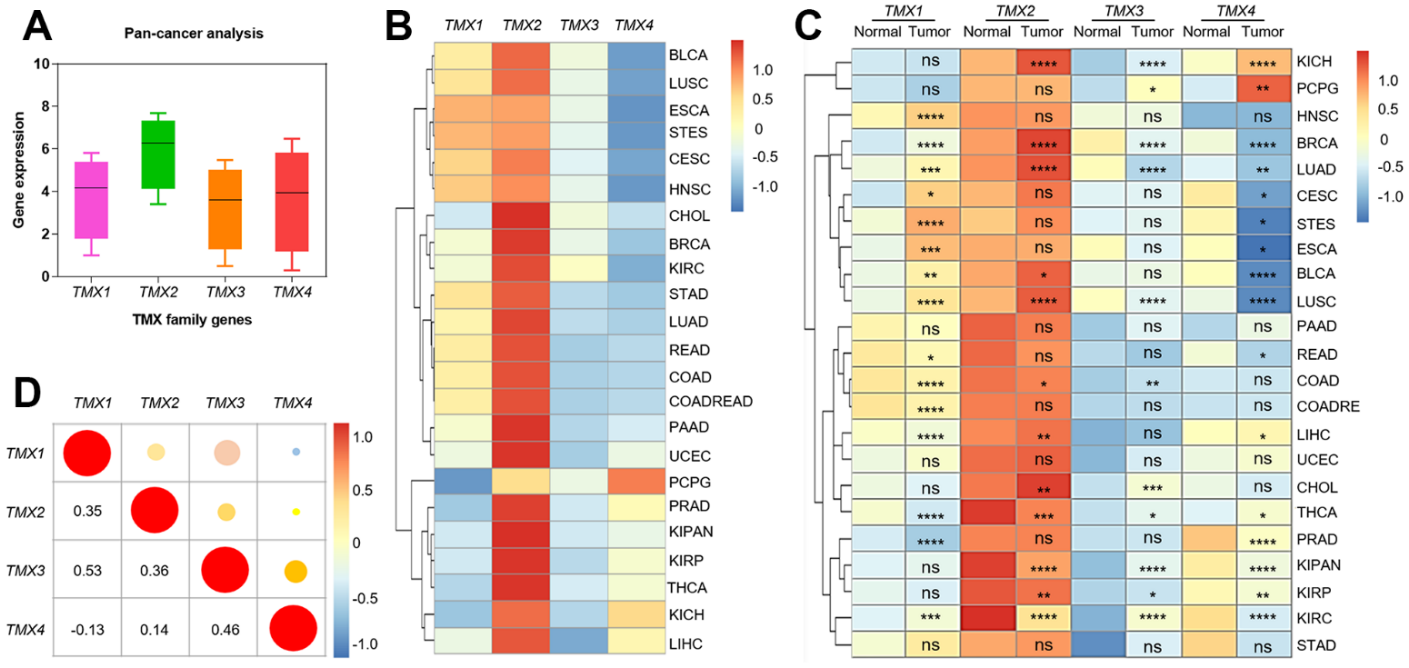
**The expression and correlation analysis of TMX family genes in different cancer types.** Box plot showed the expression of TMX family genes in pan-cancer (**A**). The expression level of TMX family genes in 23 different types of cancer (**B**). Heat map showed the difference in TMX family genes expression between 23 different types of tumors and adjacent or normal tissues (**C**). Correlation of TMX family genes expression in pan-cancer (**D**). ACC (adrenocortical carcinoma), BLCA (bladder urothelial carcinoma), BRCA (breast invasive carcinoma), CESC (cervical squamous cell carcinoma and endocervical adenocarcinoma), CHOL (cholangiocarcinoma), COAD (colorectal adenocarcinoma), COADREAD (Colon adenocarcinoma/Rectum adenocarcinoma Esophageal carcinoma), DLBC (lymphoid neoplasm diffuse large B-cell lymphoma), ESCA (esophageal carcinoma), GBM (glioblastoma multiforme), HNSC (head and neck squamous cell carcinoma), KICH (kidney chromophobe), KIRC (Kidney renal clear cell carcinoma), KIRP (kidney renal papillary cell carcinoma), KIPAN (Pan-kidney cohort), LAML (acute myeloid leukemia), LGG (brain lower grade glioma), LIHC (liver hepatocellular carcinoma), LUAD (lung adenocarcinoma), LUSC (lung squamous cell carcinoma), MESO (mesothelioma), OV (ovarian serous cystadenocarcinoma), PAAD (pancreatic adenocarcinoma), PCPG (pheochromocytoma and paraganglioma), PRAD (prostate adenocarcinoma), READ (rectal adenocarcinoma), SARC (sarcoma), SKCM (Skin Cutaneous Melanoma), STAD (stomach adenocarcinoma), STES (Stomach and Esophageal carcinoma), TGCT (testicular germ cell tumors), THCA (thyroid carcinoma), THYM (thymoma), UCEC (uterine corpus endometrial carcinoma), UCS (uterine carcinosarcoma), UVM (uveal melanoma). Red dots represent positive correlation, and blue dots represent negative correlation. * p < 0.05; * * p < 0.01; * * * p < 0.001.

To gain insights into the expression of TMX family genes across various types of cancers, we initially evaluated their expression patterns in pan-cancer using data from the TCGA and GTEx databases (Figure 1A). Overall, the highest expression of *TMX2* and the lowest expression of *TMX3* was found in pan-cancer (Figure 1B). Additionally, we also examined TMX family genes expression in both tumors and adjacent or healthy tissues. The results indicated significant differences in the expression levels of TMX family genes across different types of cancer (Figure 1C). Subsequently, we also explored the association between their expression levels in different tumors by using person correlation-coefficient analysis. We observed that *TMX1* and *TMX3* (r = 0.53) had a high positive correlation, and *TMX1* and *TMX4* (r = -0.13) showed the highest negative correlation (Figure 1D). These intriguing findings imply that TMX family genes may possess interconnected biological functions and regulatory mechanisms within the context of cancers.

### Prognostic significance of TMX family genes across pan-cancers

**Figure 2:**
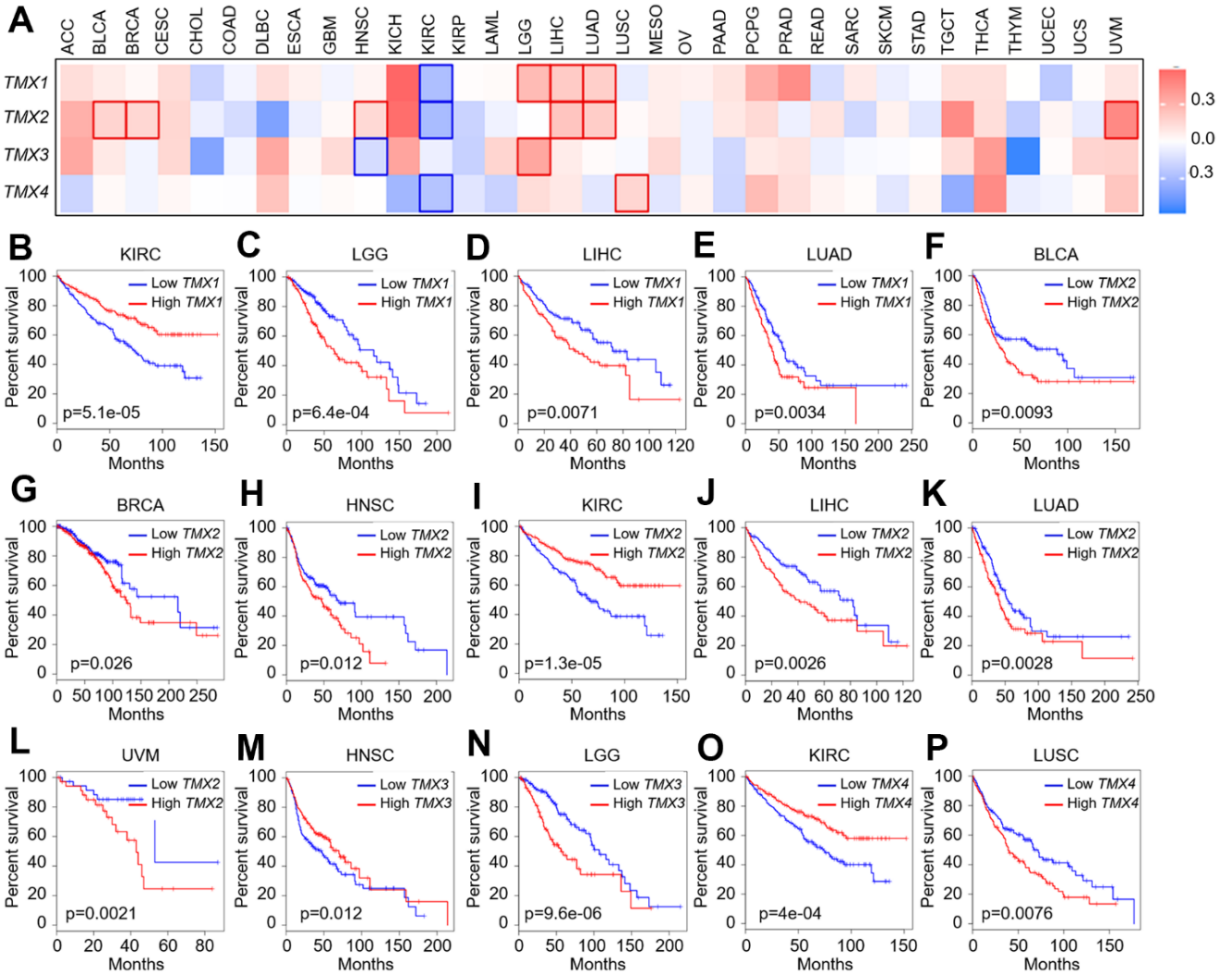
**Correlation analysis of TMX family gene expression and overall survival rate in patients with different TCGA tumor types.** Using GEPIA2 to construct a survival map of TMX family gene expression (**A**). The OS curve of *TMX1* in different tumors: KIRC (**B**); LGG (**C**); LIHC (**D**); LUAD (**E**). The OS curve of *TMX2* in different tumors: BLCA (**F**); BRCA (**G**); HNSC (**H**); KIRC (**I**); LIHC (**J**); LUAD (**K**); UVM (**L**). The OS curve of *TMX3* in different tumors: HNSC (**M**); LGG (**N**). The OS curve of *TMX4* in different tumors: KIRC (**O**); LUSC (**P**). The expression of TMX family genes was distinguished by different color lines. Red lines indicated high expression, and blue lines indicated low expression.

After exploring the expression characteristics of TMX family members on mRNA, we proceeded to assess their prognostic importance prognostic for pan-cancer in 33 cancer types. TMX family genes expression was observed to be significantly correlated with the poor prognosis of several TCGA tumors (Figure 2A). Kaplan-Meier overall survival analysis suggested that *TMX1* acted as a favorable outcome in KIRC (p = 5.1e-05, Figure 2B), and an unfavorable outcome in LGG (p = 6.4e-04, Figure 2C), LIHC (p = 0.0071, Figure 2D), and LUAD (p = 0.0034, Figure 2E). *TMX2* was a protective role in KIRC (p = 1.3e-05, Figure 2H). In contrast, *TMX2* was detrimental roles in different human cancers, including BLCA (p = 0.0093, Figure 2E), BRCA (p = 0.026, Figure 2F), HNSC (p = 0.012, Figure 2G), LIHC (p = 0.0026, Figure 2I), LUAD (p = 0.0028, Figure 2J), and UVM (p = 0.0021, Figure 2K). *TMX3* demonstrated a significant protective prognostic role in HNSC (p = 0.012, Figure 2L), and was identified as a prognostic risk factor in LGG (p = 9.6e-06, Figure 2M). *TNS4* was a protective role in KIRC (p = 4e-04, Figure 2N). Conversely, *TMX4* also was an unfavorable gene in LUSC (p = 0.0076, Figure 2O).

**Figure 3:**
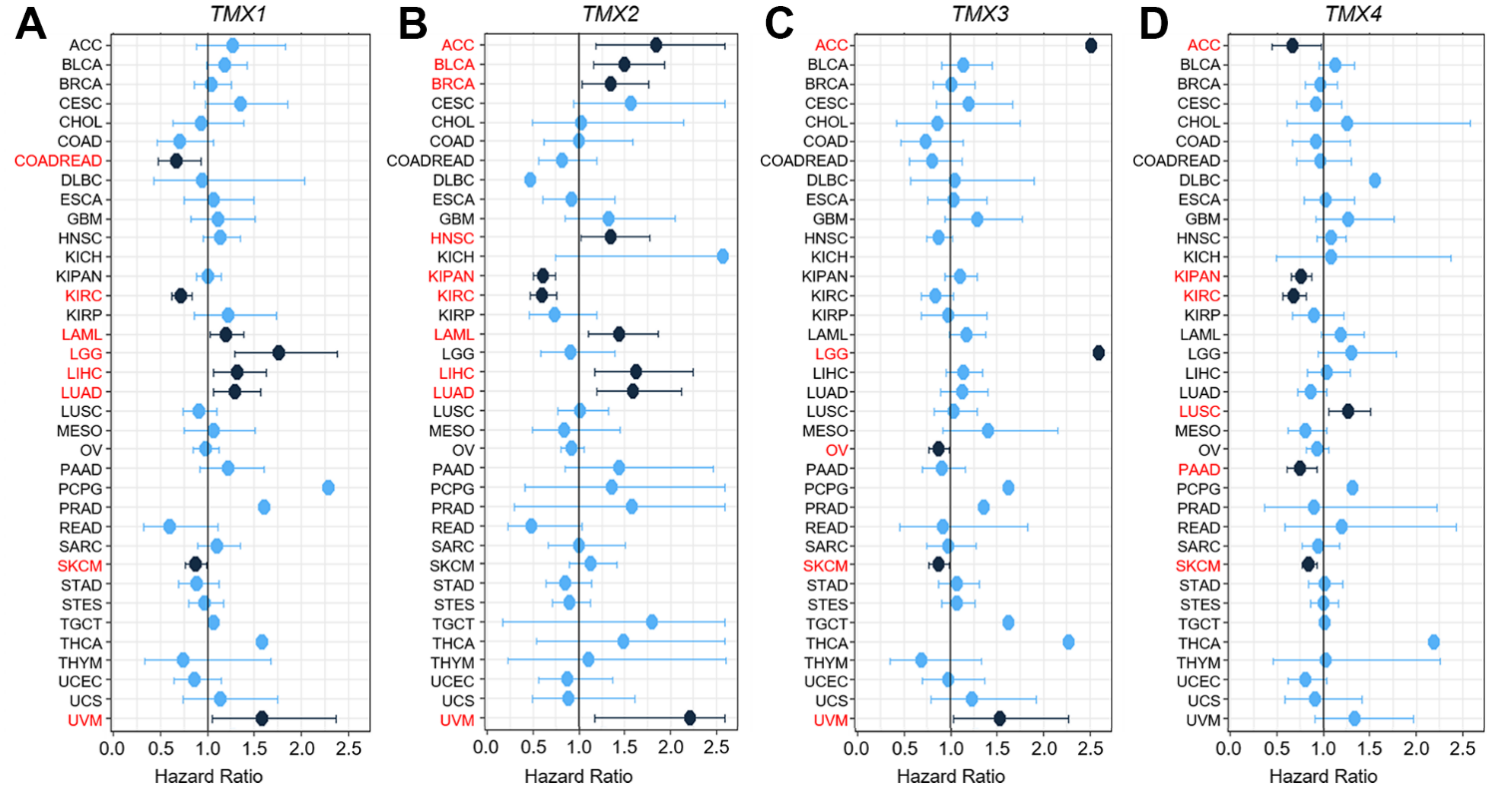
**Univariate Cox expression analysis of the relationship between TMX genes expression and OS rate in 36 different tumors.** Cox analysis of the correlation with *TMX1* expression in different cancer types (**A**). Cox regression analysis of *TMX2* expression in different types of tumors (**B**). The correlation between Cox analysis and *TMX3* expression in different cancer types (**C**). Cox regression analysis of *TMX4* expression in different types of tumors (**D**). The red words indicates that the gene is a risk factor in the corresponding tumor.

We also used univariate Cox expression analysis to assess the relationship between the expression of TMX family genes and over survival (OS) in pan-cancer from the TGCA and GTEx datasets. The findings revealed that *TMX1* played a favorable factor for prognosis in COADREAD, KIRC as well as SKCM (HR <1, p <0.05), and was a detrimental prognostic factor in LAML, LGG, LIHC, LUAD or UVM (HR >1, p <0.05, Figure 3A). *TMX2* was found to be a beneficial prognosis factor in KIRAN, KIRC (HR <1, p <0.05), and played a risk factor for ACC, BLCA, BRCA, HNSC, LAML, LIHC, LUAD or UVM (HR >1, p <0.05, Figure 3B). Our risk regression analysis revealed that *TMX3* was the low-risk gene in OV or SKCM (HR <1, p <0.05) and was a high-risk gene in ACC, LGG or UVM (HR >1, p <0.05, Figure 3C). *TMX4* emerged as a protective prognostic factor in ACC, KIPAN, KIRC, PAAD or SKCM (HR <1, p <0.05, Figure 3D), but was a detrimental prognostic factor in LUSC (HR >1, p <0.05, Figure 3D). Besides, we conducted an assessment of the relationship between TMX family genes expression and the overall survival of each cancer in different databases (including Kaplan-Meier plotter, TCGA, and TCGA and GTEx). We found several instances of contradictory data regarding the expression of TMX family genes in certain cancers (Table 1). These conflicting outcomes were due to the differences in data analysis methods and distinct biological characteristics, leading to distinct hypothetical backgrounds.

**Table 1:** The association between high expression of TMX-family genes and over survival of pan-cancer in different databases.

**Gene**	**Role**	**Kaplan-Meier plotter OS**	**TCGA OS**	**TCGA+GTEx OS**
***TMX1***	Protective	**KIRC**, LUSC, READ, STAD, THYM, UCEC	**KIRC**	COADREAD, **KIRC**, SKCM
	Detrimental	BLCA, HNSC, **LIHC**, LUAD, PAAD	LGG, **LIHC**, LUAD	LAML, LGG, **LIHC**, LUAD, UVM
***TMX2***	Protective	**KIRC**, KIRP, LUSC, OV, STAD	**KIRC**	KIPAN, **KIRC**
	Detrimental	BLCA, BRCA, HNSC, **LIHC,** LUAD, PAAD, TGCT, UCEC	BLCA, BRCA, HNSC, **LIHC**, LUAD, UVM	ACC, BLCA, BRCA, HNSC, LAML, **LIHC**, LUAD, UVM
***TMX3***	Protective	ESCA, PAAD, READ, THYM	HNSC	OV, SKCM
	Detrimental	LIHC, TGCT, THCA	LGG	ACC, LGG, UVM
***TMX4***	Protective	**KIRC**, KIRP, LUAD, PRAD, UCEC	**KIRC**	ACC, KIPAN, **KIRC**, PAAD, SKCM
	Detrimental	ESCA, LUSC	LUSC	LUSC

### Effect of TMX family genes expression on TME and stemness score

**Figure 4:**
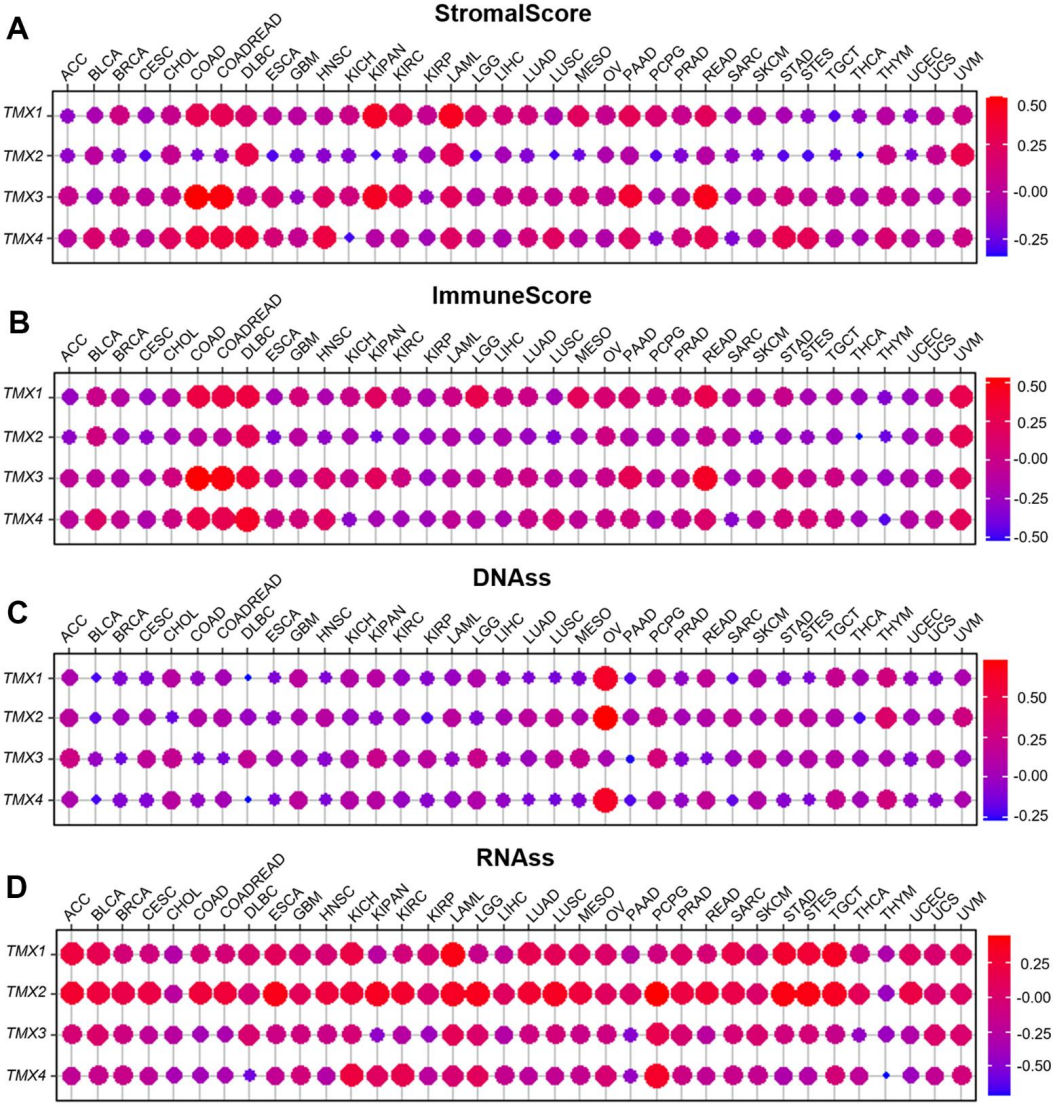
**Relationship of TMX family genes expression with tumor microenvironment and stemness score in pan-cancer.** The correlation between TMX family genes expression and StromalScore (**A**). The association with TMX family genes expression and ImmuneScore (**B**). The relationship between TMX family genes expression and DNAss (**C**). The correlation between TMX family genes expression and RNAss (**D**). Red dots represent positive correlation, and blue dots represent negative correlation.

The previous results have demonstrated the prognostic significance of TMX family genes in pan-cancer. To investigate the roles of their expression in TME, the ESTIMATE algorithm was implemented, which is presented in the form of StromalScore and ImmuneScore. The higher these scores reflect the higher abundance of matrix components and immune components in the tumor microenvironment. By estimating StromalScore and ImmuneScore in TME, we found that *TMX1*, *TMX3*, and *TMX4* exhibited a positive correlation with StromalScore (Figure 4A) and StromalScore (Figure 4B) in most cancer types, while *TMX2* expression was negatively correlated with StromalScore (Figure 4A) and ImmuneScore (Figure 4B). Furthermore, we also have observed a negative correlation of TMX family genes expression with DNAss (Figure 4C) and a positive correlation with RNAss (Figure 4D) in some type of tumors.

**Figure 5:**
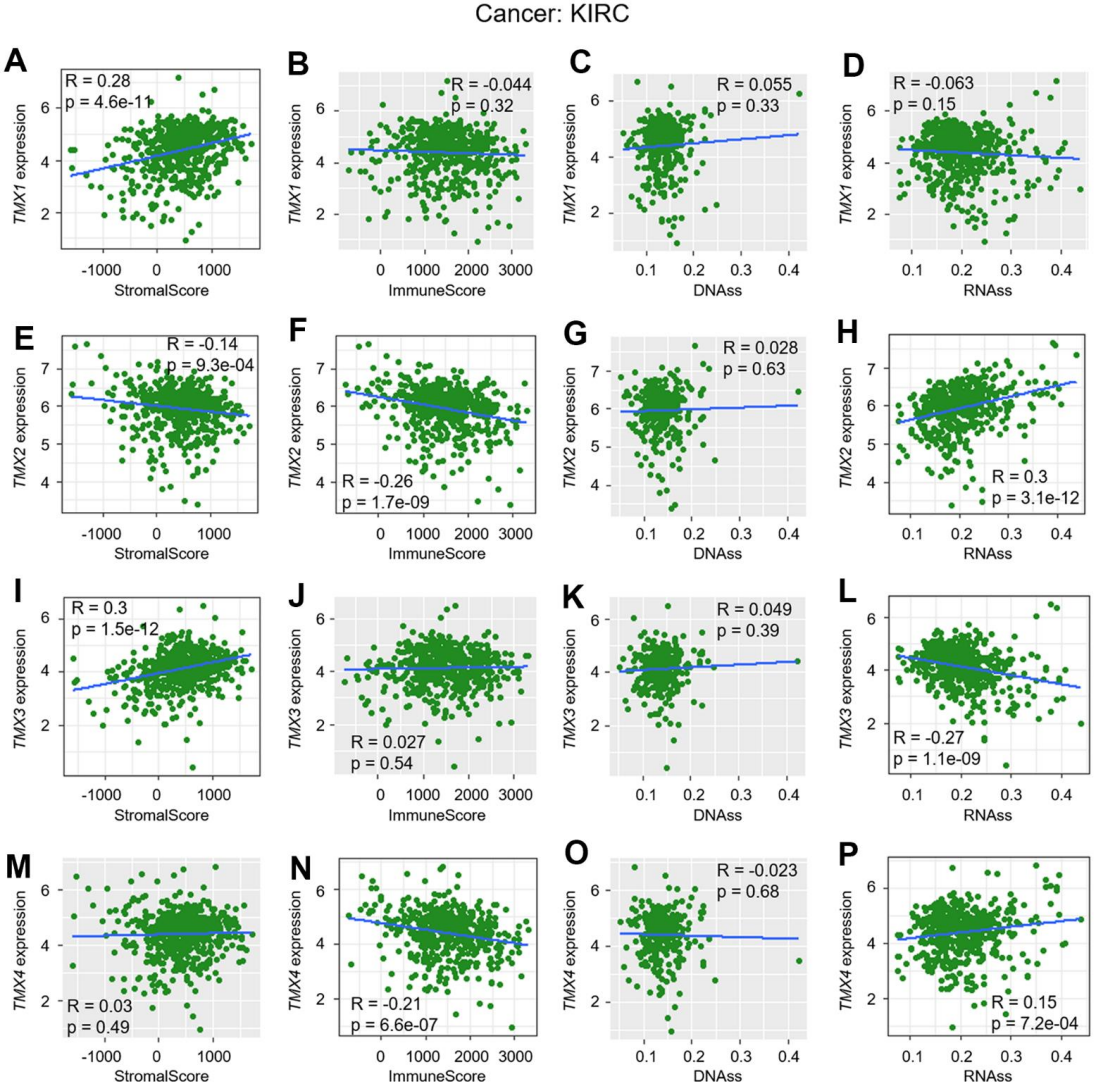
**Correlation analysis of TMX family genes expression with TME and stemness score in KIRC.** *TMX1* expression correlated with stromal score (**A**), immune scores (**B**), DNAss (**C**), and RNAss (**D**). *TMX2* expression associated with stromal score (**E**), immune scores (**F**), DNAss (**G**), and RNAss (**H**). The correlation of *TMX3* expression with stromal score (**I**), immune scores (**J**), DNAss (**K**), and RNAss (**L**). *TMX4* expression correlated with stromal score (**M**), immune scores (**N**), DNAss (**O**), and RNAss (**P**). Gray brown background indicates no correlation between the gene expression and the corresponding index (p >0.05). Light background indicates that the gene is significantly correlated with the corresponding index (p <0.05). R represents correlation value, R >0 means positive correlation, R <0 means negative correlation.

**Figure 6:**
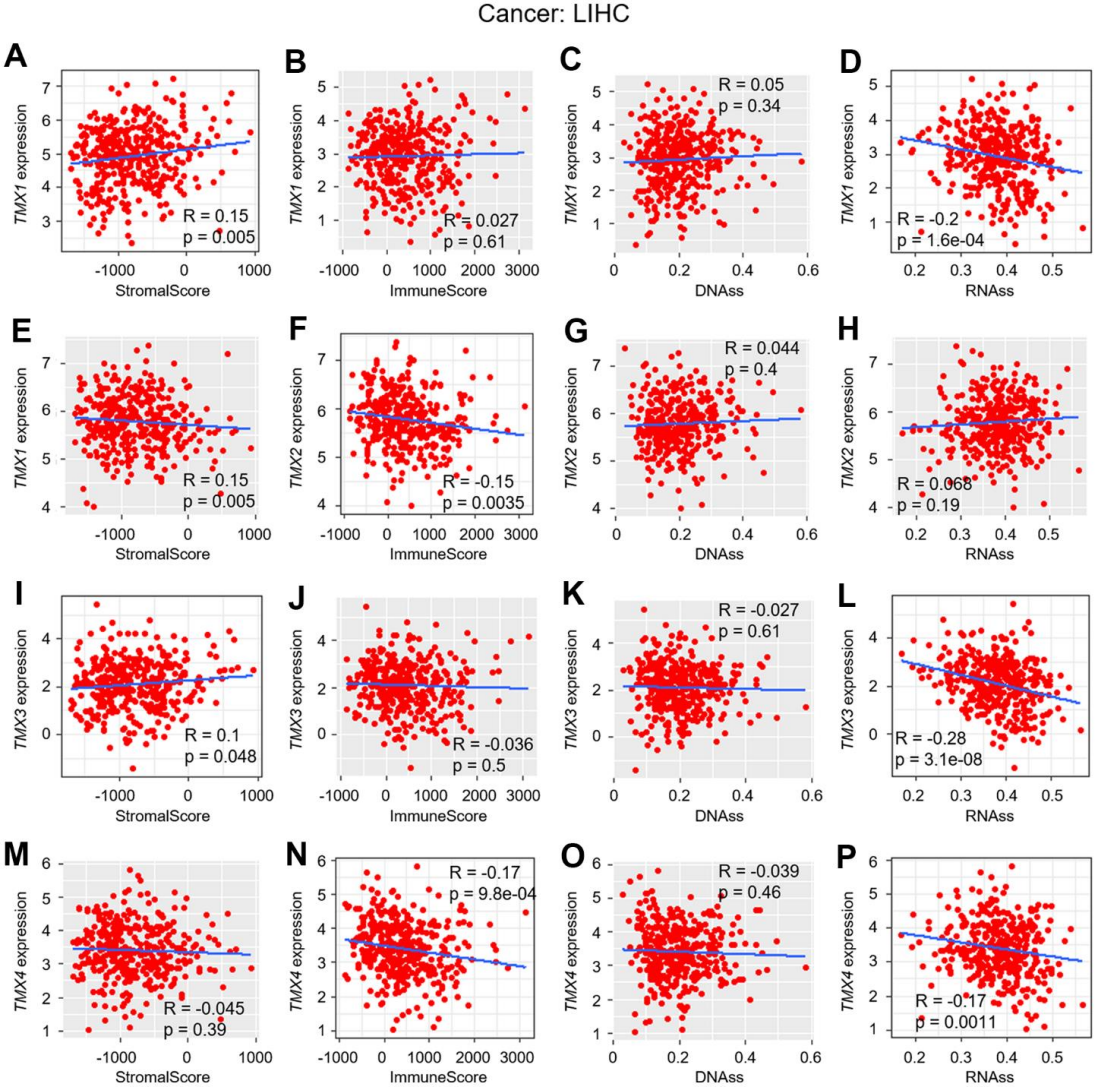
**Correlation analysis of TMX family genes expression with TME and stemness score in LIHC.** The correlation of *TMX1* expression with stromal score (**A**), immune scores (**B**), DNAss (**C**), and RNAss (**D**). *TMX2* expression correlated with stromal score (**E**), immune scores (**F**), DNAss (**G**), and RNAss (**H**). The correlation of *TMX3* expression with stromal score (**I**), immune scores (**J**), DNAss (**K**), and RNAss (**L**). *TMX4* expression correlated with stromal score (**M**), immune scores (**N**), DNAss (**O**), and RNAss (**P**). Gray brown background indicates no correlation between the gene expression and the corresponding index (p >0.05). Light background indicates that the gene is significantly correlated with the corresponding index (p <0.05). R represents correlation value, R >0 means positive correlation, R <0 means negative correlation.

In previous research, we discovered a significant association between the expression of TMX family genes and improved overall survival in KIRC and LIHC, as confirmed by multiple databases (Table 1). Building upon this, we investigated the correlation of their expression between TME, as well as stemness score in KIRC (Figure 5) and LIHC (Figure 6). Our findings revealed that *TMX1* expression was positively correlated with StromalScore in KIRC (Figure 5A). *TMX2* expression was found to be inversely correlated with KIRC StromalScore (Figure 5E) and ImmuneScore (Figure 5F), and was positively correlated with RNAss (Figure 5H). In KIRC, *TMX3* expression demonstrated a positive correlation with the StromalScore (Figure 5I) and a negative correlation with RNAss (Figure 5L). In contrast, *TMX4* expression was negatively correlated with KIRC ImmuneScore (Figure 5N) and positively correlated with RNAss (Figure 5P). We also observed that *TMX1* expression had a positive correlation with LIHC StromalScore (Figure 6A), and a negative correlation with RNAss (Figure 6D). *TMX2* expression showed a negative correlation with ImmuneScore in LIHC (Figure 6F). *TMX3* expression had a positive correlation with LIHC StromalScore (Figure 6J), and a negative correlation with RNAss (Figure 6L). *TMX4* expression was a negative correlation with ImmuneScore (Figure 6N) and RNAss (Figure 6P) in LIHC. Taking together, we observed that TMX family genes expression was related to the composition of the immune-microenvironment and tumor stemness in some types of tumors, especially in KIRC and LIHC.

### Correlation between expression of TMX family genes and immune subtype in pan-cancer data

**Figure 7:**
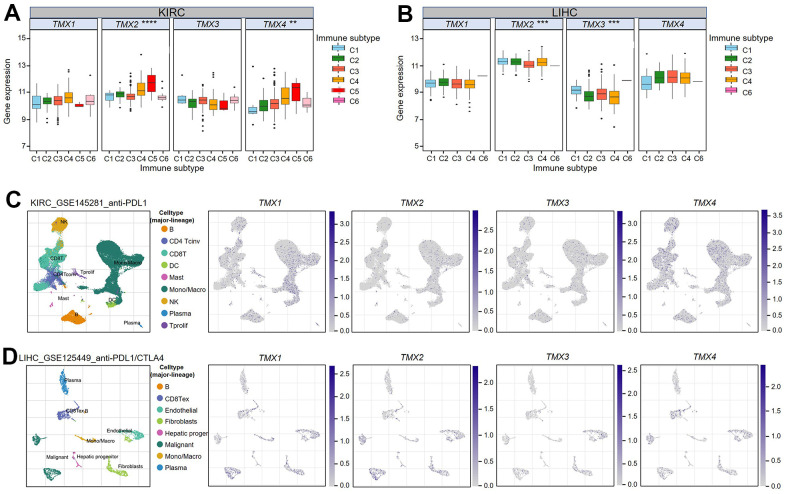
**Association of TMX family gene expression with immune infiltration subtypes in KIRC and LIHC.** One-way variance analysis was used to access the relationship between TMX family genes expression and KIRC immune infiltration subtypes (**A**). One-way analysis of variance was performed to test the correlation between TMX family genes expression and LIHC immune infiltration subtypes (**B**). UMAP plots showing cell clusters and TMX family genes expression levels in different immune cell types in KIRC (**C**). UMAP plots showing cell clusters and TMX family genes expression levels in different immune cell types in LIHC (**D**). * p < 0.05; ** p < 0.01; *** p < 0.001.

**Figure 8:**
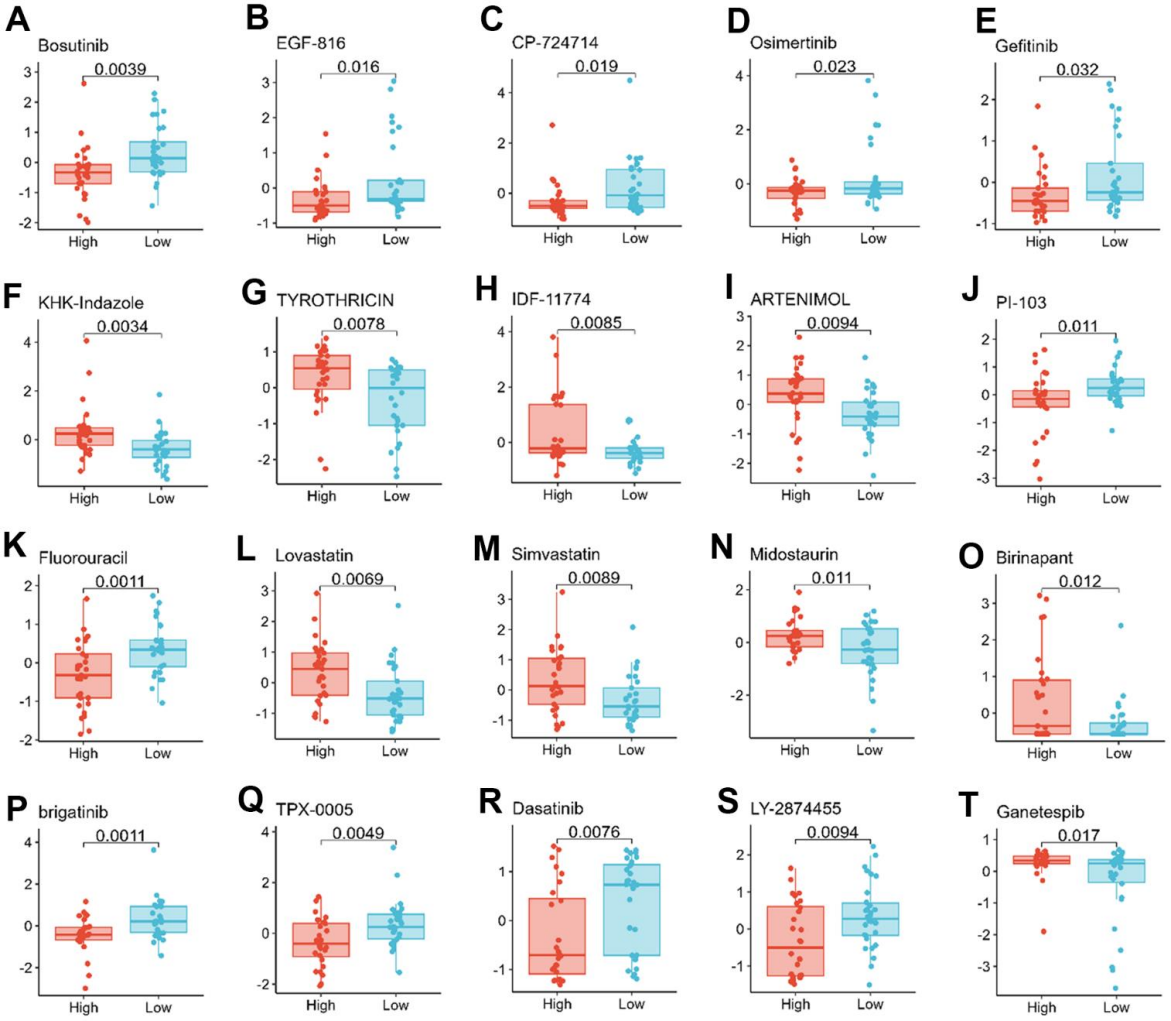
**Relationship between TMX family genes expression and drug sensitivity.** *TMX1* expression was negatively correlated with drug sensitivity of Bosutinib (**A**), EGF-816 (**B**), CP-724714 (**C**), Osimertinib (**D**), and Gefitinib (**E**). *TMX2* expression was positively correlated with the drug sensitivity of KHK-Indazole (**F**), TYROTHRICIN (**G**), IDF-11774 (**H**), ARTENIMOL (**I**), and negatively correlated with the drug sensitivity of PI-103 (**J**). *TMX3* expression was negatively correlated with the drug sensitivity of Fluorouracil (**K**), and was positively correlated with the drug sensitivity of Lovastatin (**L**), Simvastatin (**M**), Midostaurin (**N**), Binrinapant (**O**). *TMX4* expression was negatively correlated with the sensitivity of brigatinib (**P**), TPX-0005 (**Q**), Dasatinib (**R**), Ly-2874455 (**S**), but positively correlated with the drug sensitivity of Ganetespib (**T**).

Previous studies have classified human tumors into six different types of immune infiltration, including C1 (wound healing), C2 (IFN-g dominance), C3 (inflammation), C4 (lymphocyte depletion), C5 (immunologically quiet), and C6 (TGF-β dominant), and the degree of cytotoxicity increases gradually from C1 to C6 [17]. The tumor immunophenotype is a key predictor of its response to immunotherapy, and low cytotoxic immunophenotype promotes tumor development. With this in mind, we classified the tumor samples in TCGA data according to immune infiltration subtype, and subsequently analyzed their correlation with TMX family genes expression. Our analysis revealed that there are significant correlations between *TMX2* and *TMX4* with the immune subtypes of KIRC (Figure 7). In KIRC, *TNS2* expression was higher in C5 and *TMX4* expression was lower in C1 (Figure 7A). In addition, the immune subtypes of LIHC (liver hepatocellular carcinoma) showed significant associations with *TMX2* and *TMX3* (Figure 8). Specifically, the expression of *TMX2* was higher in C4, and *TMX3* expression was lower in C4 (Figure 7B). To explore the possible mechanism of TMX family genes affecting TME, the single-cell RNA-seq (scRNA) sequencing was performed. We found they were mainly expressed in monocytes/macrophages, especially in KIRC (Figure 7C) and LIHC (Figure 7D). These results suggest that TMX family genes may play important roles in immune regulation.

### TMX family genes expression and chemotherapy sensitivity

Improving drug sensitivity is an important strategy to prevent drug resistance in cancer cells [18]. Thus, further investigation the relationship of TMX family genes expression with drug sensitivity is very appropriate. We further investigated the potential correlation between the expression levels of TMX family genes and drug sensitivity in 60 human cancer cell lines (NCI-60) with over 200 chemotherapeutic agents. Among them, *TMX1* expression was negatively correlated with the drug sensitivity of Bosutinib, EGF-816, CP-724714, Osimertinib, and Gefitinib (Figure 8A–8E). *TMX2* expression was positively correlated with the drug sensitivity of KHK-Indazole, TYROTHRICIN, IDF-11774 and ARTENIMOL (Figure 8F–8I), and negatively correlated with the drug sensitivity of PI-103 (Figure 8J). During the drug sensitivity correlation analysis, we also observed that *TMX3* expression was negatively correlated with the drug sensitivity of Fluorouracil (Figure 8K), whereas it showed a significant positive correlation with the drug sensitivity of Lovastatin, Simvastatin, Midostaurin and Binrinapant (Figure 8L–8O). Conversely, *TMX4* expression showed a negative correlation with the sensitivity of brigatinib, TPX-0005, Dasatinib, and LY-2874455 (Figure 8O–8S), and acted as a positive correlation with the drug sensitivity of Ganetespib (Figure 8T). These results indicate that TMX family genes, particularly *TMX2* and *TMX4*, played an important role in determining the sensitivity of cancer cells to various chemotherapy drugs, which will be for the future in view of the TMX family genes make an important contribution to cancer treatment research.

### Association of TMX family genes with tumor metastasis and immune checkpoint genes

**Figure 9:**
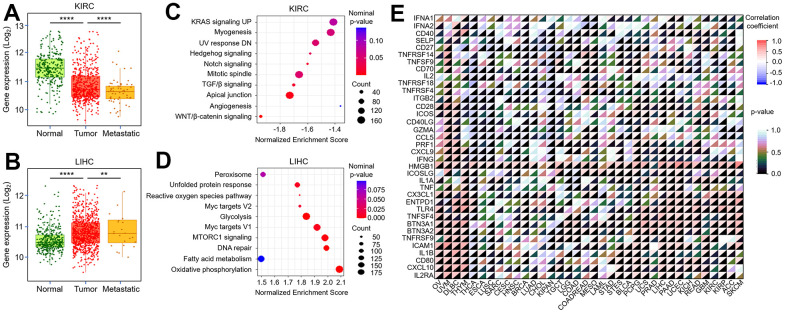
**Correlation between *TMX2* expression and tumor metastasis, immune-checkpoint-related genes.** The expression level of *TMX2* in normal, tumor and metastatic tissues of KIRC (**A**). The expression level of *TMX2* in normal, tumor and metastatic tissues of LIHC (**B**). GSEA results showing differential enrichment of signaling pathway with low *TMX2* expression phenotype in KIRC (**C**). GSEA results showing differential enrichment of signaling pathway with high *TMX2* expression phenotype in LIHC (**D**). The correlation between *TMX2* expression and immune-checkpoint-related genes in different cancer types (**E**). *p < 0.05; **p < 0.01; ***p < 0.001.

**Figure 10:**
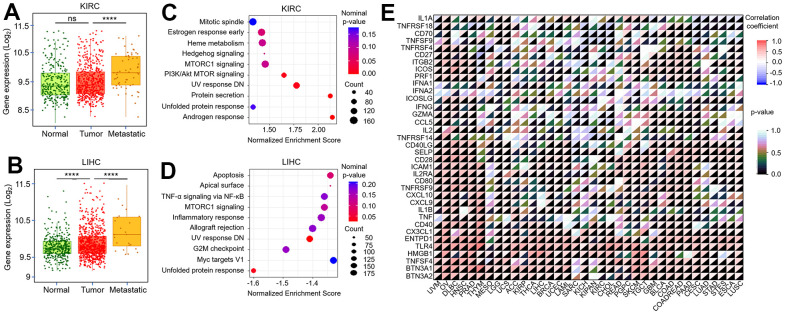
**Correlation between *TMX4* expression and tumor metastasis, immune-checkpoint-related genes.** The expression level of *TMX4* in normal, tumor and metastatic tissues of KIRC (**A**). The expression level of *TMX4* in normal, tumor and metastatic tissues of LIHC (**B**). GSEA results showing differential enrichment of signaling pathway with high *TMX4* expression phenotype in KIRC (**C**). GSEA results showing differential enrichment of signaling pathway with high *TMX4* expression phenotype in LIHC (**D**). The correlation between *TMX4* expression and immune-checkpoint-related genes in different cancer types (**E**). *p < 0.05; **p < 0.01; ***p < 0.001.

The previous results have confirmed the biological effects of TMX family genes in the tumor-microenvironment. Subsequently, we further explored the correlation of their expression with tumor metastasis. Our results found *TMX2* expression was negatively correlated with KIRC metastasis (Figure 9A), and positively correlated with metastasis in LIHC (Figure 9B). We also observed *TMX4* expression had a positive correlation with the metastasis of KIRC (Figure 10A) and LIHC (Figure 10B). In order to better evaluate the signaling pathways activated by *TMX2* and *TMX4* in KIRC and LIHC, GSEA was implemented. We revealed signaling pathways that are significantly enriched in low *TMX2* expression phenotype (Figure 9C) and high *TMX4* expression phenotype (Figure 9D) in KIRC, respectively. In addition, we also explored the signaling pathways that are significantly enriched in high *TMX2* (Figure 10C) and *TMX4* (Figure 10D) expression phenotypes in LIHC. These findings suggest that *TMX2* and *TMX4* may regulate the proliferation, invasion and metastasis of KIRC and LIHC through the above pathways. Furthermore, we also investigated the correlation of *TMX2* and *TMX4* expression with immune-checkpoint-related stimulator genes in pan-cancer. Our results demonstrated that they were positively or negatively correlated with these immune checkpoint genes (Figures 9E, 10E).

### TMX2 promotes epithelial-mesenchymal transition (EMT) in liver cancer

**Figure 11:**
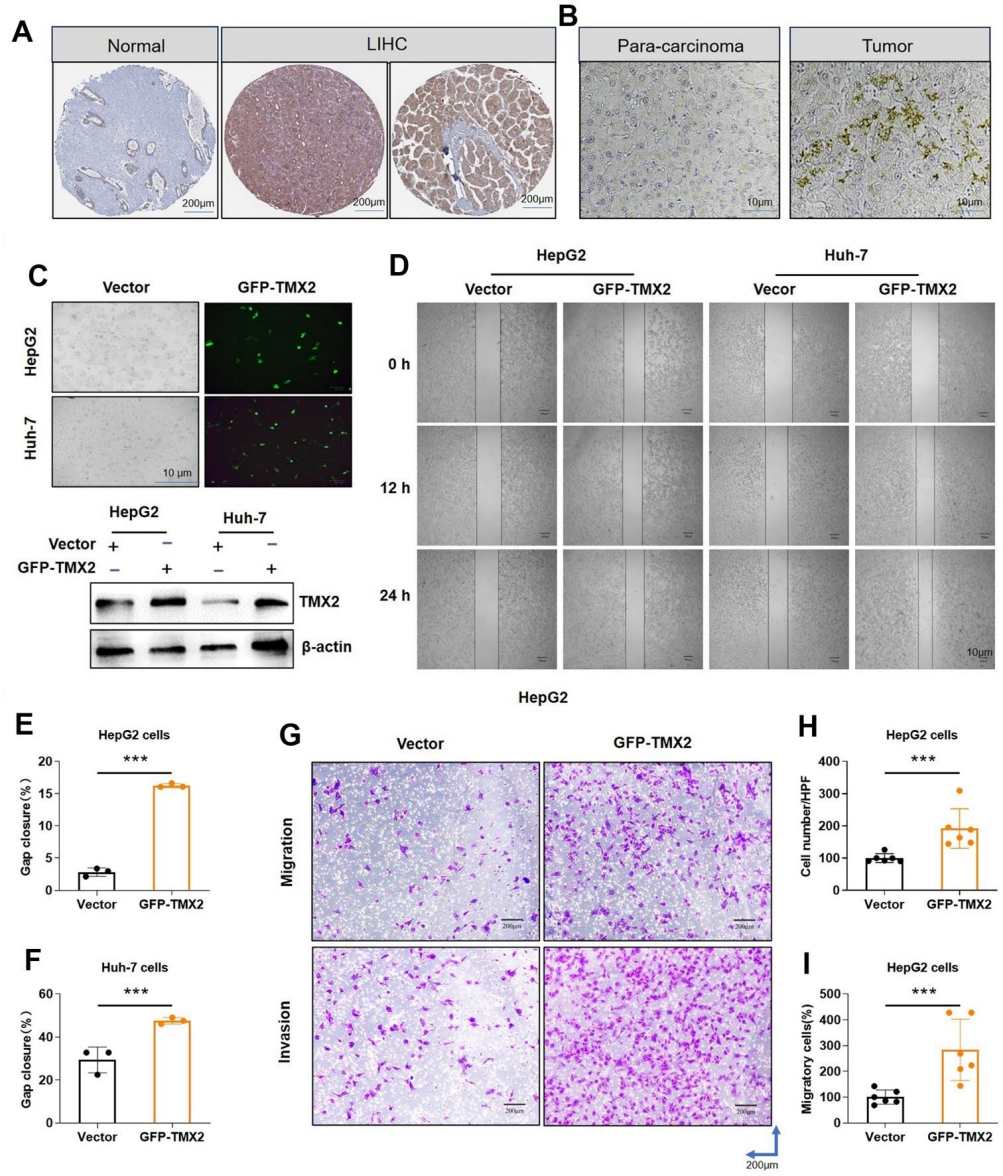
**TMX2 overexpression promotes the migration and invasion of liver cancer cells *in vitro*.** Based on the HPA database, representative immunohistochemical staining of TMX2 in normal and tumor tissues of LIHC (**A**). Immunohistochemical analysis of TMX2 expression intensity in liver cancer patient (**B**). Transfection analysis of TMX2 (**C**). Microscopic observations were recorded at 0, 12, and 24 h after scratching the surface of a confluent layer of the indicated HepG2 and Huh-7 cells (**D**). Quantitative analysis of wound healing percentage in HepG2 cells (**E**). Quantitative analysis of wound healing percentage in Huh-7 cells (**F**). The effects of TMX2 on cell migration and invasion were examined by transwell assays in HepG2 cells (**G**). Quantitative analysis of cell migration in HepG2 cells (**H**). Quantitative analysis of cell invasion in HepG-2 cells (**I**). *p < 0.05, **p < 0.01, ***p < 0.001.

We previously found that *TMX2* expression was positively correlated with the metastasis of LIHC. By analyzing the actual protein expression in human tumor tissues, we found that the TMX2 protein is more strongly expressed in tumor tissues compared to adjacent normal tissues (Figure 11A). Subsequently, we found similar results in the clinical collection of case tissues by immunohistochemical staining (Figure 11B). To further investigate the roles of TMX family members in hepatocellular carcinoma cells invasion, transfection experiments were performed. We first overexpressed TMX2 plasmid with GFP green fluorescence in HepG2 and Huh-7 cells (Figure 11C). In comparison to the control group, TMX2 overexpression caused significantly faster wound healing percentage in HepG2 (Figure 11D, 11E) and Huh-7 cells (Figure 11D, 11F). Subsequently, transwell experiment was performed to investigate the cells invasion ability. The results showed that TMX2 up-regulation promoted the migration (Figure 11G, 11H) and invasion (Figure 11G, 11I) of HepG2 cells compared to the control group.

**Figure 12:**
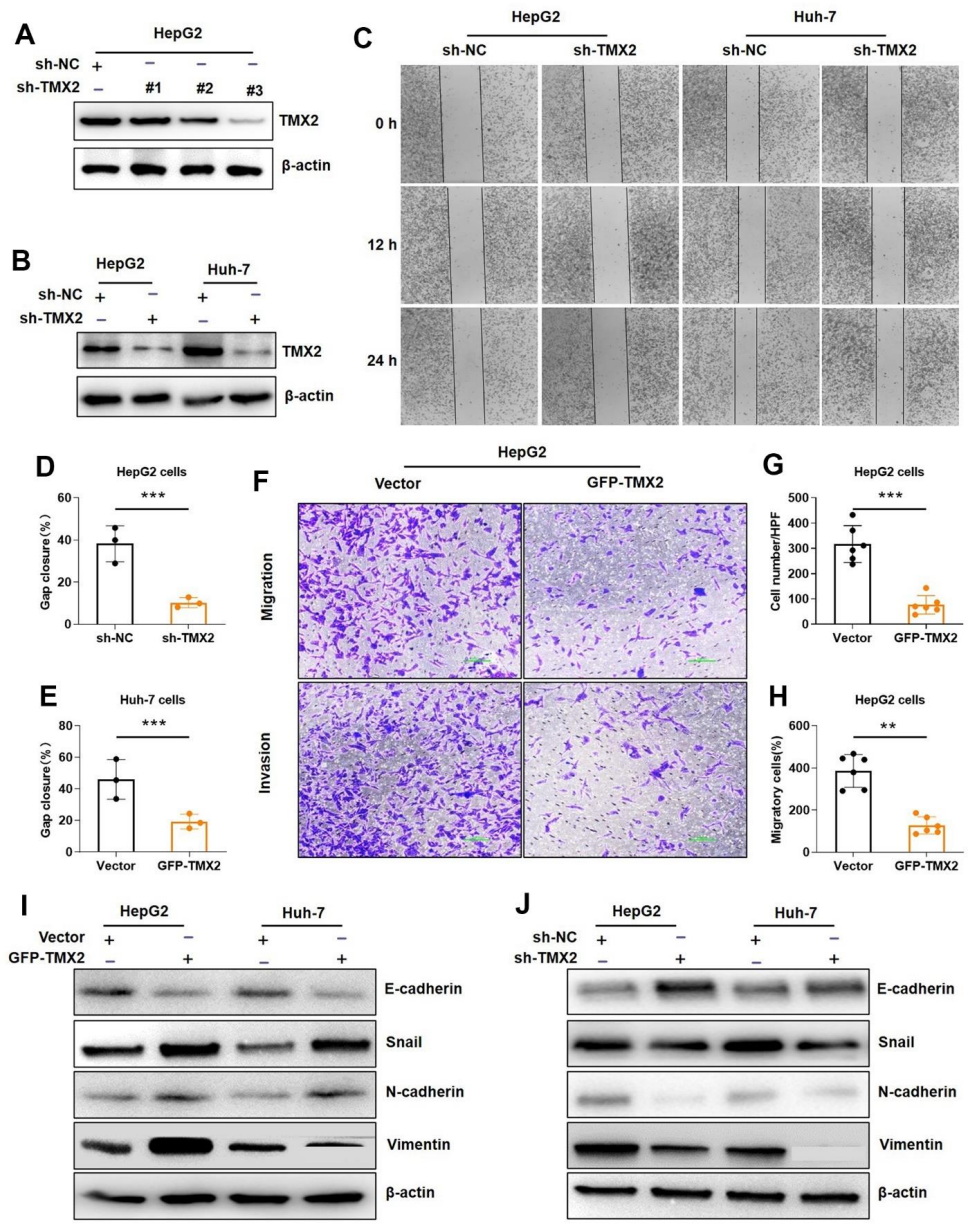
**TMX2 promotes epithelial-mesenchymal transition (EMT) in liver cancer cells.** Verification of sh-TMX2 (**A**). Transfection analysis of TMX2 (**B**). Microscopic observations were recorded at 0, 12, and 24 h after scratching the surface of a confluent layer of the indicated HepG2 and Huh-7 cells (**C**). Quantitative analysis of wound healing percentage in HepG2 cells (**D**). Quantitative analysis of wound healing percentage in Huh-7 cells (**E**). The effects of TMX2 on cell migration and invasion were examined by transwell assays in HepG2 cells (**F**). Quantitative analysis of cell migration in HepG2 cells (**G**). Quantitative analysis of cell invasion in HepG-2 cells (**H**). Western blot analysis of EMT marker expression in TMX2-overexpressing HepG2 and Huh-7 cells (**I**). Western blot analysis of EMT marker expression in TMX2-deficient HepG2 and Huh-7 cells (**J**). *p < 0.05, **p < 0.01, ***p < 0.001.

To further investigate the biological roles of TMX2 knock-down on invasion and migration of hepatoma cell lines (HepG2 and Huh-7), we also transfected shRNA plasmid targeting TMX2 into cells to knockdown endogenous TMX2. Among the different plasmids, we found that plasmid 3# had a better effect, and it was also selected for subsequent knockdown experiments. (Figure 12A, 12B). Compared with the control group, TMX2 knockdown caused significantly slower wound healing percentage in HepG2 (Figure 12C, 12D) and Huh-7 cells (Figure 12C, 12E). Similarly, TMX2 knockdown attenuated the migration (Figure 12F, 12G) and invasion (Figure 12F, 12H) of HepG2 cells. Additionally, we also found the exogenous expression of TMX2 in HepG2 and Huh-7 cells led to alterations resembling EMT. This was indicated by the decrease in levels of E-cadherin, along with increases in levels of Snail, N-cadherin, and Vimentin (Figure 12I). Conversely, silencing TMX2 in HepG2 and Huh-7 cells resulted in the opposite effects (Figure 12J).

## DISCUSSION

The application of multi-omics techniques in tumor research has found that the specific signaling pathways commonly acquire activating mutations among multiple cancers. Pan-cancer analyses of different tumors provide comprehensive insights into tumor biology and cancer molecular phenotypes, which contributes to identify genomic changes that probably play important roles in carcinogenic phenotypes and also points the way to finding biomarkers for targeted therapies [19]. With the deepening of investigation on the genetic characteristics of tumors, numerous studies have been conducted to explore targeted molecular biomarkers and functional characterization in pan-cancer [20]. In this study, based on TCGA and GTEx databases, we analyzed the expression of TMX family (*TMX1*, *TMX2*, *TMX3*, and *TMX4*) genes in 23 different cancer types. Compared with healthy tissues, *TMX1* showed a significant up-regulation in 10 cancer types. *TMX2* was significantly over-expressed in 4 cancer types. *TMX3* expression was decreased in 3 cancer types and significantly upregulated in 2 cancer types. *TMX4* was significantly down-regulated in 6 cancer types and up-regulated in 4 cancer types. These findings contribute to determine whether TMX family genes are possible target genes for oncogenic and their relevance to the development of cancer immunotherapy.

As a constituent of the thioredoxin family members located in the endoplasmic reticulum, TMX family genes possess protein disulfide isomerase, which play a crucial role in protein folding [21, 22]. Many disease-related mutations or abnormalities occur in membrane intrinsic proteins. Alterations in the expression of TMX family genes can impact protein folding or transport, resulting in protein assembly errors, which specifically affect protein function, especially when mutations occur in the endoplasmic reticulum [23]. Although emerging evidence have explored the correlation with gene mutations and human cancer progression [24], only a few changes have been found to significantly affect cancer development [25]. Survival analysis revealed TMX family genes expression was a significantly association with poor prognosis in some tumors. The down-expression of *TMX1*, *TMX2*, and *TMX3* shortened the overall survival time of KIRC, while the increased expression of *TMX1* and *TMX2* predicted a poor prognosis for OS in LIHC and LUAD. Moreover, the results of the Cox regression model analysis demonstrated that TMX family genes could serve as a significant risk factor for pan-cancer prognosis, particularly in KIRC and LIHC. However, it has not been investigated for the mechanism that TMX family genes expression impacts the prognosis of human cancer. In the current study, our findings provide a foundation for more detailed investigation of the biological roles of TMX family genes in tumors. Besides regulating the tumor microenvironment and immune cell infiltration, we reveal the association of their expression levels with tumor metastasis and immune checkpoint-related genes.

Previous studies have performed immunogenomic research on over 1000 tumor cases from 33 cancer types from the TCGA [26]. From this analysis, six distinct immune subtypes were determined, namely wound healing (C1), IFN-g dominant (C2), inflammation (C3), lymphocyte depletion (C4), immune quiescence (C5), and TGF-β dominance (C6). Among them, C2 exhibited the highest IFN-g characteristics, C3 was mainly immune activation as well as related to immune infiltration, and C5 was dominated by M2 macrophages [27]. The tumor immunophenotype provides comprehensive insights into the infiltration of immune cells in various types of cancer and contribute to identify critical predictors of early cancer diagnosis and immunotherapy responses [28]. In this study, our aim was to examine the relationship of TMX family genes expression with immune subtypes in pan-cancer, especially in KIRC and LIHC. These findings indicate that TMX family genes may exert important roles in immune regulation.

Drug resistance is a main reason leading to cancer treatment failure, which limits the selection and usage of cancer drugs [29]. Using NCI-60 cell line dataset, we explored the relationship of TMX family genes expression with drug sensitivity. Our findings reveal that increased expression of TMX family genes is associated with resistance to certain FDA-approved chemotherapeutic drugs. Specifically, we observed a negative correlation between *TMX1* expression and the sensitivity to Bosutinib, EGF-816, CP-724714, Osimertinib and Gefitinib. *TMX2* expression exhibited a positive correlation with the sensitivity of KHK-Indazole, TYROTHRICIN, IDF-11774 and ARTENIMOL, and negatively correlated with PI-103. In the correlation analysis of drug sensitivity, we also discovered a negative correlation between *TMX3* expression and the drug sensitivity of Fluorouracil, and a positive correlation with Lovastatin, Simvastatin, Midostaurin, and Binrinapant. *TMX4* expression was negatively correlated with the sensitivity of brigatinib, TPX-0005, Dasatinib, and Ly-2874455, but positively correlated with the drug sensitivity of Ganetespib. Additionally, we also conducted an analysis on the relationship between TMX family genes expression and immune-checkpoint stimulation-related-genes. Collectively, our results appear that TMX family genes may participate in modulating tumor cell sensitivity or resistance to drug treatment, and therefore hold promise as potential therapeutic targets for overcoming drug-induced resistance or enhancing drug sensitivity as a complement to existing therapies.

The phenotypic and genetic characteristics of gene expression in tumors are closely related to the activation of specific signaling pathways [30]. In certain cases, cancer relies on these features for its proliferation, invasion, and metastasis [31]. It is, therefore, crucial to further investigate the relationship between TMX family genes expression and tumor metastasis. In this study, we found that low *TMX2* expression significantly promoted KIRC metastasis, and it exhibited a positive correlation with LIHC metastasis. High *TMX4* expression promoted the metastasis of KIRC and LIHC. To gain a better understanding of the biological mechanism of TMX family genes in cancers, we conducted KEGG signaling pathway enrichment analysis on TMX family genes (*TMX2* and *TMX4*) with different expression phenotypes in KIRC and LIHC, respectively. This analysis is vital as it provides valuable insights into the regulatory mechanism of TMX family genes in tumors. Our results showed that the Wnt / β-catenin signaling, Apical junction, and TGF-β signaling were significantly enriched in low *TMX2* expression phenotypes in KIRC, while Oxidative phosphorylation, DNA repair, MTORC1 signaling, Myc targets V1, Glycolysis, Myc targets V2. Reactive oxygen species pathway, and unfolded protein response were significantly enriched in high *TMX4* expression phenotype. In LIHC, the Androgen response, Protein secretion, UV response DN, PI3K / Akt MTOR signaling were significantly enriched in high *TMX2* expression phenotype, while the high expression phenotype of TMX4 was significantly associated with enrichment of Unfolded protein response and UV response DN signaling pathway. These observations, coupled with the present study’s findings, effectively substantiate the hypothesis that the TMX family genes has a key effect in tumor progression regulation by modulating the aforementioned signaling pathways.

Despite systematic elucidation of the roles of TMX family genes in pan-cancer from various perspectives such as expression pattern, poor prognosis, tumor immune microenvironment, stemness score, immune subtype and drug sensitivity, there are still certain shortcomings in the current research. Primarily, this study focused on bio-informatics exploration of TMX family genes expression and survival prognosis in pan-cancer data, lacking some biological experiment. Moreover, although TMX family genes expression is linked to human malignancy heterogeneity and clinical survival, it remains unclear whether TMX family genes affect clinical survival through immune signaling pathways. Hence, it is imperative that future studies are conducted to explore TMX family genes expression and tumor heterogeneity, which contribute to provide additional insights into the mechanisms underlying this issue.

## CONCLUSIONS

In summary, based on datasets from different databases, we explored the possible correlation between TMX family genes expression and poor prognosis, tumor microenvironment, drug resistance in pan-cancer. Furthermore, our research has unveiled a connection between TMX family genes expression is associated and tumor metastasis and immune checkpoints. These discoveries underscore the clinical significance of TMX family genes as potential biomarkers for cancer prognosis, and provide valuable perspectives for enhancing the efficiency of immunotherapy.

## MATERIALS AND METHODS

### Pan-cancer data mining and analysis

Using the UCSC Xena online-database (https://xenabrowser.net/datapages/), we collected RNA sequencing datasets (HTSeq - FPKM), clinical information, immunoinfiltration subtypes and stemness score for different human cancer types in Genotype-Tissue Expression (GTEx) and The Cancer Genome Atlas Program (TCGA) databases. We obtained a total of 18,397 cases of 36 different cancer types, adjacent tissues as well as healthy tissues. For pan-cancer analysis, we extracted TMX family genes expression, including *TMX1*, *TMX2*. *TMX3*, *TMX4*. Subsequently, the R-package “pheatmap” was performed to compare the expression data of TMX family genes between cancerous tissues and adjacent tissues. For statistical analysis, we employed the Wilcoxon rank-sum test in R software. R-packages “corrplot” and “ggplot2” were used for correlation analysis and visualization analysis, respectively [32]. The levels of significance were denoted as *p < 0.05, **p < 0.01, ***p < 0.001, ****p < 0.0001.

### Prognosis and Cox regression analysis in pan-cancer

We utilized various databases, including GEPIA2 (http://gepia.cancer-pku.cn/index.html) and Kaplan-Meier plotter (http://kmplot.com/analysis/), to investigate the effects of TMX family genes on pan-cancer prognosis. GEPIA2 is a cancer data mining website that primarily relies on TGCA and GTEx projects [33]. We used a cutoff value of 50% as the expression threshold to distinguish between high- or low-expression groups, and the overall survival (OS) maps of TMX family genes in 33 different tumor types were generated by the survival map module of GEPIA2. These survival maps were then compared with the Logrank value, and the log rank p < 0.05 was considered statistically significant. We performed COX univariate regression analysis to determine the role of TMX family genes expression levels in the risk of human pan-cancer. For visual analysis, we utilized the R package “forestplot” to create a forest-plot.

### Tumor microenvironment and stemness score analysis

The ESTIMATE algorithm was performed to assess the relationship of TMX family genes expression with the extent of immune cell infiltration and stromal cells in various cancer types by calculating StromalScore and ImmuneScore. The Pearson correlation coefficient was employed to examine the relationship between tumor microenvironment and TMX family genes expression. The correlation of TMX family genes expression with DNA stemness score (DNAss) and RNA stemness score (RNAss) were analyzed using R-packages “cor.Test”, “ggpubr” and “limma”. A positive correlation was indicated by R > 0, while a negative correlation was indicated by R < 0. A p-value of less than 0.05 was considered to be a significant difference.

### Immune infiltration subtypes analysis

The immune subtype data for different tumor types were obtained from the UCSC Xena database. To measure immune infiltration in the tumor microenvironment, six immune subtypes (C1~C6) were defined [27]. Analysis of variance model was used to evaluated the relationship between TMX family genes expression and immune infiltration subtypes in tumor microenvironment. R-package “ggplot2” was utilized for visual analysis. Statistical differences were evaluated using Wilcox’s test, and p <0.05 was considered statistically significant. *p <0.05, **p <0.01, ***p <0.001, ****p <0.0001.

### Single-cell sequencing analysis

Visualization of TME using a public online database called Tumor Immune Single-Cell Hub (TISCH2, http://tisch.comp-genomics.org/). TMX family genes expression at the single-cell level in the KIRC_GSE145281 and LIHC_GSE125449 datasets was visualized using the “dataset” module.

### Drug sensitivity analysis

We collected the RNAseq expression data and sensitivity processing data of different drugs from the CellMiner™ online database (http://discover.nci.nih.gov/cellminer/home.do). This database, known as NCI-60, includes 60 distinct human cancer cell lines and is widely regarded as a valuable resource for cancer research groups [34]. To ensure the validity of analysis results, we selected the drug with clinical trials and FDA approval as the study object in drug sensitivity analysis. For visual analysis, we utilized the R-package “ggplot2.”

### Tumor metastasis and immune checkpoint genes analysis

The expression difference of TMX family genes in normal tissues, tumor tissues and metastases were investigated by TNM mapper (https://tnmplot.com/analysis/). For immune checkpoint analysis, the Pearson correlation-coefficient methods were performed to evaluate the relationship of TMX family members expression with immune checkpoint genes. R packages “pheatmap” and “ggplot2” were used for data analysis and result visualization.

### Gene-set enrichment analysis (GSEA)

To examine how the expression of TMX family gene expression impacts tumor metastasis, GSEA (version 4.0.3) was implemented. The gene sets database of “h.all.v7.2.symbols.gmt” was used to compare the differences in the phenotypes of different genes [35]. The nominal p-value and false-discovery-rate (FDR) q-value were employed to assess group differences. p <0.05 and FDR <0.25 were considered statistically significant. The R packages “AnnotationHub”, “org.Hs.eg.db”, “clusterProfiler” and “dplyr” were used for enrichment analysis.

### Cell culture and transfection

HepG2 and Huh-7 cells were obtained from ATCC (American Type Culture Collection) and were maintained in DMEM (Gibco, C11995500BT) supplemented with 10% FBS (Gibco, 10091148) and 1% Penicillin-Streptomycin (Gibco, 15140122) at 37° C with 5% CO_2_. Plasmid transfection was performed on cells inoculated in 6-well plates or 12-well plates, following the Lipofectamine 3000 transfection reagent (Invitrogen, L3000015) protocol. To synthesize the *TMX2* sequence, the full-length *TMX2* sequence (NM_030755.5, available on NCBI) was used and then sub-cloned into a pEGFP-N1 vector (Invitrogen, Shanghai, China). The control consisted of the empty pEGFP-N1 vector Three short hairpin RNAs (shRNAs) targeting TMX2 (TMX2 shRNA#1: 5′-GCTGAAAGTAAAGAAGGAACA-3′; TMX2 shRNA#2: 5′-AGGACTGAGTGGACGGTTTAT-3′; and TMX2 shRNA#3: 5′-CGTGCCAAGCAATAAGATTTA-3′) and negative control (shNC: 5′-CCTAAGGTTAAGTCGCCCTCG-3) were cloned into the lentiviral pLKO.1_puro vector (Public Protein/Plasmid Library), respectively.

### Immunoblotting and immunohistochemical staining

The cells were collected and the proteins were extracted. After that, the proteins were blotted onto polyvinylidene fluoride (PVDF) after using 10% SDS-PAGE separation. The membrane was subsequently blocked using 5% skim milk in Tris-buffered saline with Tween 20 (TBST) before incubation with target antibodies (4° C overnight). These specific antibodies include anti-TMX2 (PA5-102865, Invitrogen), anti-E-cadherin (3195S, CST), N-Cadherin (13116S, CST), Snail (3879S, CST), Vimentin (5741S, CST), and β-actin (4967, CST). The membranes were then incubated with the secondary antibody for 2 h at room temperature (20~30° C) before the blot samples was imprinted using an Easysee Western Blot Kit (Transgene, Alsace, France). From September 2023 to October 2023, a total of 7 liver hepatocellular carcinoma (LIHC) specimens and corresponding para-carcinoma tissue or normal specimens were obtained from the Affiliated Hospital of Traditional Chinese Medicine of Southwest Medical University. Immunohistochemical analysis was conducted according to the previous article [36], and the antibody was obtained from Invitrogen (PA5-116023). All specimens were clinically and histologically diagnosed as LIHC and were stained in a blinded manner by pathologists. Additionally, we also received the immunohistochemical images of TMX2 from the Human Protein Atlas (https://www.proteinatlas.org/) online-database.

### Wound healing assay

Cells were initially plated on 6-well plates with a density of 6 × 10 ^6^ cells / well, and transfected with empty vector plasmid and *TMX2* plasmid the next day. On the third day, after the successful transfection of the plasmid was observed, and subsequently the monolayer cells were scratched using a 200 μL pipette tip. Afterward, the floating cells were then thoroughly washed with PBS. Photographed under a microscope. After that, the cells were cultured for 12 h and 24 h, respectively, and photographed.

### Transwell assay

For the migration experiment, we added 5 × 10 ^4^ HepG2 cells to the upper chamber which was filled with serum-free medium. and also added 500 μL of DMEM medium with 20 % serum to the lower chamber. Regarding the invasion assay, we prepared the upper chamber by coating it with matrigel diluted in serum-free medium at a ratio of 1:8 before inoculating the cells. All the remaining steps remained the same as migration assay. After allowing the cells to incubate for a period of 48 h, the cells passing through the bottom of the lower chamber were fixed and stained, and finally photographed and counted.

### Statistical analysis

The results were expressed as the means ± standard error (SE) of a minimum of 3 independent experiments performed in triplicate. To conduct the variance analysis, we utilized the GraphPad Prism 8.0 software package. The t-test was applied for analysis of paired samples, and the ANOVA analysis was performed to detect unpaired samples. Statistical significance was defined as *p < 0.05, **p< 0.01, ***p< 0.0001.

### Data availability statement

All original data from this study have been deposited in the online database (https://xena.ucsc.edu/), and the whole visualizations utilized in this paper have also been included. Additionally, other data employed to support the findings of this study can be requested from the corresponding author. It is important to note that the authors confirm the accessibility of all original data in this research from a public database.
