# Correction for: TMX family genes and their association with prognosis, immune infiltration, and chemotherapy in human pan-cancer

**DOI:** 10.18632/aging.205990

**Published:** 2024-06-30

**Authors:** Na Luo, Zhiqiang Mei, Qiqi Zhang, Hong Tang, Runlan Wan, Anni Deng, Xiaopan Zou, Chaoxiang Lv

**Affiliations:** 1 The Research Center for Preclinical Medicine, Southwest Medical University, Luzhou 646000, Sichuan, China; 2 School of Basic Medical Sciences, Southwest Medical University, Luzhou 646000, Sichuan, China; 3 Degree Office, The Graduate School of Southwest Medical University, Luzhou 646000, Sichuan, China; 4 Department of Pathology, Affiliated Hospital of Southwest Medical University, Luzhou 646000, Sichuan, China; 5 Department of Oncology, The Affiliated Hospital of Southwest Medical University, Luzhou 646000, Sichuan, China; 6 Department of Pediatrics, Southwest Medical University, Luzhou 646000, Sichuan, China; 7 Breast and Thyroid Surgery, Renmin Hospital, Jilin University, Changchun 130024, Jilin, China

**Keywords:** pan-cancer, thioredoxin, tumor microenvironment, immune infiltration subtypes, epithelial-mesenchymal transition

**This article has been corrected:** The authors recently found that they inadvertently used adjusted instead of the original images for the “Vimentin” western blots shown in **Figure 12I** and **12J**: Western blot analysis of EMT marker expression in TMX2-overexpressing HepG2 and Huh-7 cells (**I**), and Western blot analysis of EMT marker expression in TMX2-deficient HepG2 and Huh-7 cells (**J**). The authors provided original uncropped images of all western blots and corrected the mistake with the original “Vimentin” images. These corrections have no impact on the main conclusion. The authors apologize for any confusion or inconvenience caused by this error.

The corrected version of **Figure 12** is provided below.

**Figure 12:**
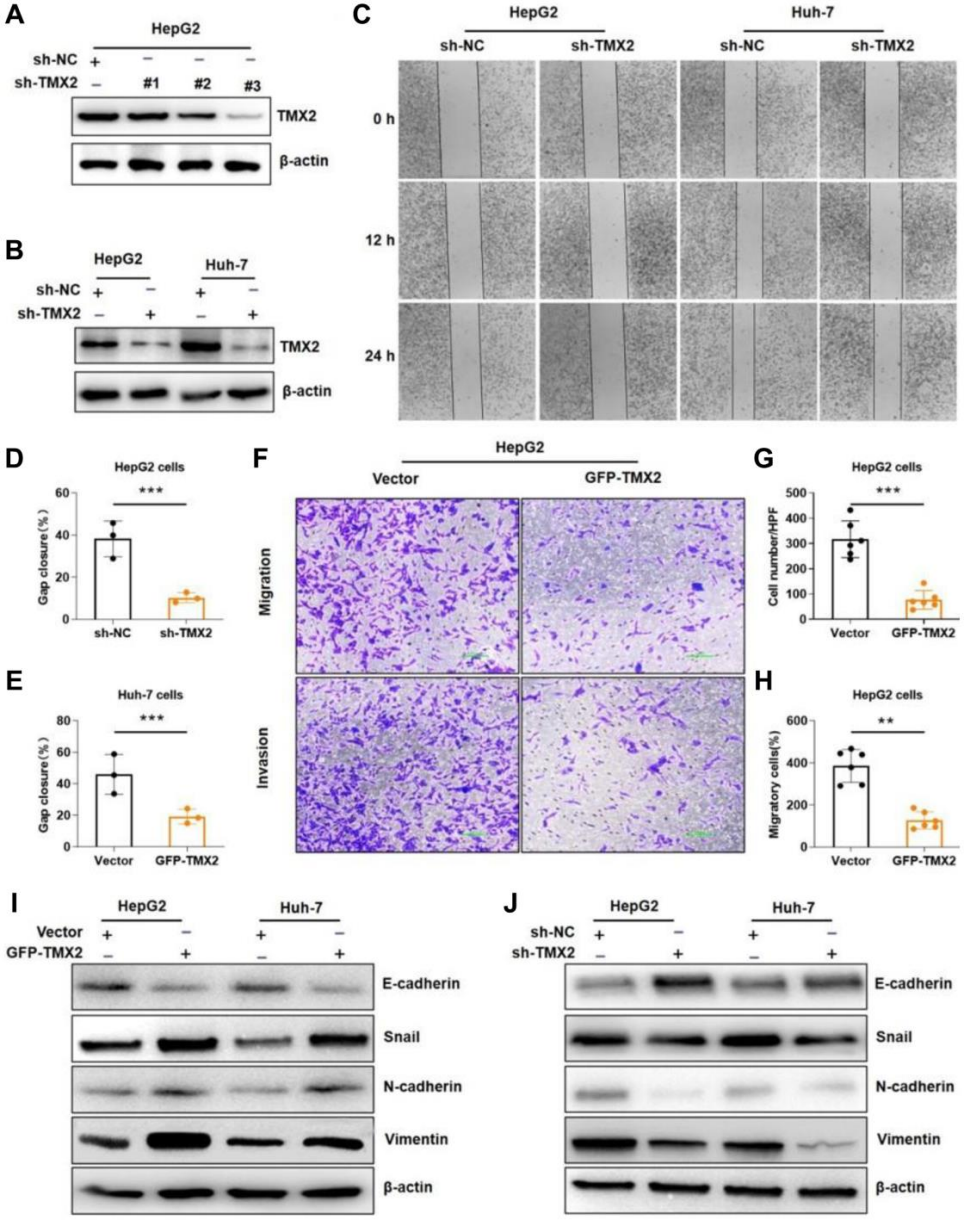
**TMX2 promotes epithelial-mesenchymal transition (EMT) in liver cancer cells.** Verification of sh-TMX2 (**A**). Transfection analysis of TMX2 (**B**). Microscopic observations were recorded at 0, 12, and 24 h after scratching the surface of a confluent layer of the indicated HepG2 and Huh-7 cells (**C**). Quantitative analysis of wound healing percentage in HepG2 cells (**D**). Quantitative analysis of wound healing percentage in Huh-7 cells (**E**). The effects of TMX2 on cell migration and invasion were examined by transwell assays in HepG2 cells (**F**). Quantitative analysis of cell migration in HepG2 cells (**G**). Quantitative analysis of cell invasion in HepG-2 cells (**H**). Western blot analysis of EMT marker expression in TMX2-overexpressing HepG2 and Huh-7 cells (**I**). Western blot analysis of EMT marker expression in TMX2-deficient HepG2 and Huh-7 cells (**J**). ^*^p < 0.05, ^**^p < 0.01, ^***^p < 0.001.

